# The Omega-3 Fatty Acid Docosahexaenoic Acid Modulates Inflammatory Mediator Release in Human Alveolar Cells Exposed to Bronchoalveolar Lavage Fluid of ARDS Patients

**DOI:** 10.1155/2015/642520

**Published:** 2015-08-02

**Authors:** Paolo Cotogni, Antonella Trombetta, Giuliana Muzio, Marina Maggiora, Rosa Angela Canuto

**Affiliations:** ^1^Anesthesiology and Intensive Care, Department of Medicine, S. Giovanni Battista Hospital, University of Turin, Via A.M. Dogliotti 14, 10126 Turin, Italy; ^2^Department of Medical Sciences, University of Turin, Via A.M. Dogliotti 14, 10126 Turin, Italy; ^3^Department of Experimental Medicine and Oncology, University of Turin, Corso Raffaello 30, 10125 Turin, Italy

## Abstract

*Background*. This study investigated whether the 1 : 2 *ω*-3/*ω*-6 ratio may reduce proinflammatory response in human alveolar cells (A549) exposed to an *ex vivo* inflammatory stimulus (bronchoalveolar lavage fluid (BALF) of acute respiratory distress syndrome (ARDS) patients). *Methods*. We exposed A549 cells to the BALF collected from 12 ARDS patients. After 18 hours, fatty acids (FA) were added as docosahexaenoic acid (DHA, *ω*-3) and arachidonic acid (AA, *ω*-6) in two ratios (1 : 2 or 1 : 7). 24 hours later, in culture supernatants were evaluated cytokines (TNF-*α*, IL-6, IL-8, and IL-10) and prostaglandins (PGE_2_ and PGE_3_) release. The FA percentage content in A549 membrane phospholipids, content of COX-2, level of PPAR*γ*, and NF-*κ*B binding activity were determined. *Results*. The 1 : 2 DHA/AA ratio reversed the baseline predominance of *ω*-6 over *ω*-3 in the cell membranes (*P* < 0.001). The proinflammatory cytokine release was reduced by the 1 : 2 ratio (*P* < 0.01 to <0.001) but was increased by the 1 : 7 ratio (*P* < 0.01). The 1 : 2 ratio reduced COX-2 and PGE_2_ (*P* < 0.001) as well as NF-*κ*B translocation into the nucleus (*P* < 0.01), while it increased activation of PPAR*γ* and IL-10 release (*P* < 0.001). *Conclusion*. This study demonstrated that shifting the FA supply from *ω*-6 to *ω*-3 decreased proinflammatory mediator release in human alveolar cells exposed to BALF of ARDS patients.

## 1. Introduction

Acute respiratory distress syndrome (ARDS) is a form of acute diffuse lung injury associated with a predisposing risk factor, characterized by inflammation leading to increased pulmonary vascular permeability and loss of aerated lung tissue [1]. According to the Berlin definition, the acute phase of this syndrome is manifested by the early onset of respiratory failure (within 1 week of a known clinical insult or new/worsening respiratory symptoms) [2]. The main characteristic of the clinical syndrome is hypoxemia; specifically, each subcategory of ARDS (mild, moderate, and severe) is defined by mutually exclusive ranges of the ratio between partial pressure of arterial oxygen (PaO_2_) and fraction of inspired oxygen (FiO_2_) (200 mm Hg < PaO_2_/FIO_2_ ≤ 300 mm Hg, 100 mm Hg < PaO_2_/FIO_2_ ≤ 200 mm Hg, and PaO_2_/FIO_2_ ≤ 100 mm Hg, resp.) [2]. Mortality from severe ARDS in the 1970s was as high as 85–90% but, from 2000, it decreased to 20–40% [3].

In the ARDS early phase, the alveolar space is characterized by alveolar infiltration with neutrophils and macrophages, and both are able to release inflammatory cytokines with an accumulation of both proinflammatory and anti-inflammatory cytokines [4, 5]. Many cytokines were detected at elevated levels in bronchoalveolar lavage fluid (BALF) in patients with ARDS, that is, tumor necrosis factor (TNF)-*α*, interleukin (IL)-1*β*, IL-6, and IL-8. The key role of a hyperinflammatory response, mainly characterized by the overproduction of proinflammatory cytokines, in the progression of the lung damage is well-documented [6, 7]. Moreover, many studies reported an increased mortality in patients who have elevated proinflammatory cytokine concentrations (TNF-*α*, IL-6, and IL-8) in the BALF at the onset of ARDS or persistent increased concentrations [8, 9]. Similarly, low levels of anti-inflammatory cytokines (i.e., IL-10 and IL-1 receptor antagonist) in the BALF in the ARDS early phase are associated with an increased mortality [10, 11]. Thus, the balance between proinflammatory and anti-inflammatory cytokines is of greater importance because the degree of cytokine imbalance is a contributing element to ARDS severity [12].

Over the last years, there has been an improving understanding of polyunsaturated fatty acid (PUFA) pathophysiology and several mechanisms for the interaction between PUFAs and inflammation or immune response have been demonstrated [13–16]. Indeed, after PUFA supply (diet or enteral and parenteral administration), many cell properties and related functions are modified, mainly the inflammatory and immunity responses [13]. Briefly, omega- (*ω*-) 3 PUFAs are more regarded as anti-inflammatory agents, whereas *ω*-6 PUFAs are regarded as proinflammatory agents. Recently, it was speculated that *ω*-3 PUFAs may be involved in the resolution of inflammation [14].

The discovered ability of *ω*-3 PUFAs to downregulate several different responses of the inflammatory process had suggested that these PUFAs might be used not exclusively as nutrients but mainly as pharmacological agents. Since the 1990s, many editorials have stressed the possibility to modulate the inflammatory response in acute lung injury (ALI) or ARDS patients using *ω*-3 PUFAs as drugs (the so-called “pharmaconutrition”) [17–19]. Subsequently, a great number of studies with cell or animal models have been carried out with the aim to demonstrate the efficacy of fish oil or their main active components (i.e., eicosapentaenoic acid (EPA) and docosahexaenoic acid (DHA)) in modifying the inflammatory responses [20–22]. Likewise, there have been several randomized controlled trials of enteral [23–28] or parenteral [5, 29–31] administration of fish oil-enriched nutrition formulas in mechanically ventilated patients with ALI, ARDS, or sepsis.

In a previous study [32], we demonstrated that shifting the PUFA supply from *ω*-6 PUFA (i.e., arachidonic acid (AA)) to *ω*-3 PUFA (i.e., DHA) significantly reduced the release of proinflammatory cytokines in human alveolar cells undergoing lipopolysaccharide (LPS) challenge. Moreover, we found that, in the presence of a DHA/AA ratio with a predominance of AA, there was a cytokine balance more oriented towards a proinflammatory response than with LPS alone. The aim of this study was to investigate whether in human alveolar cells the 1 : 2 DHA/AA ratio was effective in reducing proinflammatory response induced by an* ex vivo* inflammatory stimulus such as BALF of ARDS patients.

## 2. Materials and Methods

### 2.1. Bronchoalveolar Lavage Fluid Collection

The BALF was collected from ARDS patients—requiring mechanical ventilation and BAL for clinical purposes—within 24 hours after endotracheal intubation and stored at −80°C as previously described [33]. The selection of ARDS patients was carried out as previously described [34]. The institutional review board approved the study.

### 2.2. Cell Culture and Treatment

The human lung carcinoma cell line A549 (ATCC, Rockville, MD, USA) was used. A549 are alveolar epithelial cells with type II pneumocyte properties. The A549 were cultured in HAM-F12 K medium (Sigma-Aldrich, St Louis, MO, USA) and treated as previously described [35].

BALF was added 24 hours after seeding, while LPS (400 *μ*g/mL) was used as positive control. After 18 hours, A549 cells were treated with 50 *μ*M of 1 : 2 DHA/AA ratio (DHA 17 *μ*M plus AA 33 *μ*M) or 50 *μ*M of 1 : 7 DHA/AA ratio (DHA 6.5 *μ*M plus AA 43.5 *μ*M) for 24 hours. PUFAs and LPS from* Escherichia coli* 055:B5 were obtained from Sigma-Aldrich.

### 2.3. ELISA

At the experimental end point (i.e., after 24 hours), cytokine contents were measured in BALF (i.e., IL-1*β*, TNF-*α*, IL-6, and IL-8) and in culture supernatants (i.e., TNF-*α*, IL-6, IL-8, and IL-10) via enzyme-linked immunosorbent assay (ELISA). Cytokine kits were purchased from Euroclone (Paignton-Devon, UK). Prostaglandins E_2_ and E_3_ (PGE_2_ and PGE_3_) release was measured in culture supernatants via ELISA. Prostaglandin kits were purchased from MyBioSource (San Diego, CA, USA). The assays were performed according to the manufacturer's instructions.

### 2.4. Fatty Acid Percentage Content in Membrane Phospholipids

At the experimental end point, the fatty acid (FA) percentage content was determined as previously described [36]. Briefly, total lipids were extracted by the method of Folch et al. [37] and separated by thin-layer chromatography using n-heptane : isopropylether : formic acid (90 : 60 : 3) as solvent. Phospholipids bands (deposition line) were scraped, extracted, and used for FA determination. FA methyl esters were prepared following the method of Metcalfe et al. [38] and separated by gas-liquid chromatography (CP 9002 Chrompack International B.V., Middelburg, Netherlands). Internal standard (methyl heptadecanoate) was added to each preparation to determine recovery.

### 2.5. Preparation of Total Cell Lysate for Western Blot Analysis

Total cells lysates were obtained as previously described [35]. Briefly, cells were sonicated in HCMF buffer containing 1% Triton, 0.1% SDS, 2 mM Calcium Chloride (CaCl_2_), 100 *μ*g/mL phenylmethylsulfonyl fluoride (PMSF), and 1 *μ*g/mL leupeptin at an intermediate setting (output *≅* 3) using a Branson Sonifier 250 (VWR Scientific, OH, USA). Lysates were cooled on ice for 3–5 min and the sonicating–cooling cycle was repeated for a total of 3 cycles.

### 2.6. Preparation of Cytoplasmic and Nuclear Extracts for Western Blot Analysis

At the experimental end point, culture media were collected and stored at −80°C until cytokine concentration evaluation with ELISA and cells were lysed for cytoplasmic and nuclear protein fractions extraction. Briefly, cells were lysed into buffer A (10 mM HEPES pH 7.9, 10 mM KCl, 0.1 mM EDTA) added with 1 mM DTT, 0.5 mM PMSF, 5 *μ*L of 10 *μ*g/*μ*L of aprotinin, leupeptin, and pepstatin A and 40 *μ*L/mL of IGEPAL 10%. Cell lysates were centrifuged with a Microfuge at 15,000 rpm × 3 min at 4°C. Supernatants cytoplasmic fractions were collected and stored at −80°C until use. Nuclei pellets were lysated, for 2 h at 4°C, in buffer B (20 mM HEPES, pH 7.9, 0.4 M NaCl, 1 mM EDTA, and 10% glycerol) added with 1 mM DTT, 0.5 mM PMSF, 5 *μ*L of 10 *μ*g/*μ*L of aprotinin, leupeptin, and pepstatin A. Nuclei lysates were centrifuged with a Microfuge at 15,000 rpm × 5 min at 4°C. Nuclei fractions were collected and stored at −80°C until use. HEPES, KCl, NaCl, EDTA, glycerol, DTT, PMSF, aprotinin, leupeptin, pepstatin A, and IGEPAL were purchased from Sigma-Aldrich.

### 2.7. Western Blot Analysis

Protein concentrations in the cell, nuclei, and cytoplasmic extracts were measured using the Protein Assay Kit 2 according to the manufacturer's instructions (Bio-Rad Laboratories, Hercules, CA, USA). Thirty micrograms of proteins from the cell or nuclei fractions were separated by SDS-polyacrylamide gel electrophoresis using 10% polyacrylamide gel. Proteins were then transferred onto polyvinylidene difluoride membranes (Immobilon-P, Millipore, MA, USA). The membranes were blocked overnight using 5% nonfat milk in 50 mM Tris/150 mM HCl (pH 7.5) containing 0.1% Tween 20 (TBS/Tween). After three 5-min rinses with TBS/Tween, membranes were probed with polyclonal anti-PPAR*γ*, anti-COX-2, anti-p65 NF-*κ*B, or anti-I*κ*B*α* (Santa Cruz Biotechnology, Heidelberg, Germany) for 1 h at room temperature. After three 5-min rinses with TBS/Tween, horseradish peroxidase- (HRP-) conjugated secondary antibodies goat anti-rabbit or goat anti-mouse IgG (Santa Cruz Biotechnology) were applied for 1 h at room temperature. Protein bands were visualized using a chemiluminescence detection system (Immun-Star HRP; Bio-Rad Laboratories, Hercules, CA, USA). To normalize protein signals, stripped PVDF membranes were reprobed with monoclonal anti-*β*-actin (Sigma-Aldrich) for cytosolic and cell fractions or with polyclonal anti-lamin A/C (Santa Cruz Biotechnology).

### 2.8. Statistical Analysis

Data were expressed as mean ± standard deviation. Multiple comparisons were carried out using one-way analysis of variance (ANOVA), followed by Bonferroni post hoc test. SPSS statistical Software, version 14 (SPSS Inc, Chicago, IL, USA), was used for analyses. Significance was defined as *P* < 0.05.

## 3. Results and Discussion

### 3.1. BALF Cytokine Pattern in ARDS Patients

**Figure 1:**
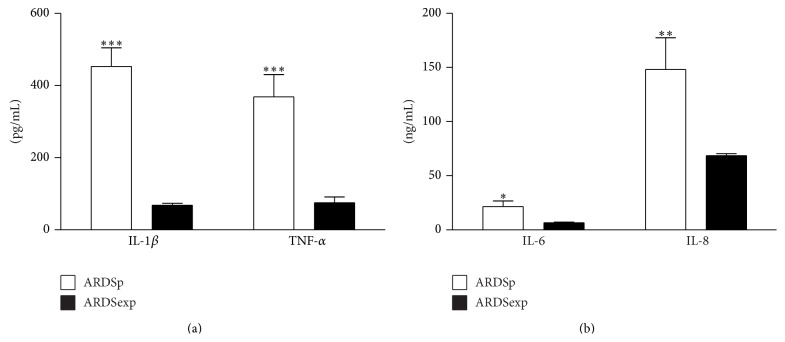
BALF cytokine pattern in ARDS. (a) IL-1*β* and TNF-*α* proinflammatory cytokine content in BALF collected from pulmonary (ARDSp, white bars) and extrapulmonary (ARDSexp, black bars) ARDS patients. (b) IL-6 and IL-8 cytokine content in BALF collected from pulmonary (ARDSp, white bars) and extrapulmonary (ARDSexp, black bars) ARDS patients. The results are expressed as picograms (pg) or nanograms (ng) of cytokines per mL as indicated. Data are presented as mean ± standard deviation of 12 independent determinations (*n* = 7 ARDSp and *n* = 5 ARDSexp). BALF, bronchoalveolar lavage fluid; ARDS, acute respiratory distress syndrome; IL, interleukin; TNF, tumor necrosis factor. ^*^ *P* < 0.05 ARDSp versus ARDSexp. ^**^ *P* < 0.01 ARDSp versus ARDSexp. ^***^ *P* < 0.001 ARDSp versus ARDSexp.

The BALF was collected from twelve adult ARDS patients (9 males and 3 females; age 56 ± 4 yrs; pneumonia *n* = 7 and sepsis *n* = 5; Simplified Acute Physiology Score (SAPS) II 42 ± 18). Proinflammatory cytokines IL-1 *β* and TNF-*α* were significantly higher in primary pneumonia patients (pulmonary ARDS, ARDSp) than in patients with ARDS originating from sepsis (extrapulmonary ARDS, ARDSexp) (Figure 1(a)). This was also observed for IL-6 and, in particular, IL-8 content (Figure 1(b)).

It has been extensively documented that in BALF of ARDS patients the content of proinflammatory cytokines is markedly elevated [12]. TNF-*α* and IL-1*β* are the main proinflammatory cytokines and they are important in driving the initial lung inflammatory response principally through the stimulation of other cytokines (i.e., IL-6 and IL-8). IL-6 occupies a critical place in modulating ARDS inflammatory response, while chemokine IL-8 is the major neutrophil chemotactic factor into the alveolar space and it is an early marker for the development of ARDS [10]. In particular, a high content of IL-6 and IL-8 is correlated with the progression of lung injury as well as with a poor outcome [8, 9].

There are many etiologic risk factors for ARDS, which are generally divided into those associated with direct injury (ARDSp) to the lung and those that cause indirect lung injury (ARDSexp) in the setting of a systemic process [17, 39, 40]. Indeed, experimental and clinical studies showed little overall differences in the inflammatory responses between direct and indirect lung injury categories. Thus, identification of the risk factor leading to ARDS in the single patient, regardless of its direct or indirect nature, is rather useful to guide treatment for the underlying disease causing ARDS [1]. Moreover, clinical data supported the theory that an overaggressive and persistent patient inflammatory response, rather than the condition causing lung injury, is the most important factor affecting survival in ARDS patients [41].

### 3.2. BALF of ARDS Patients Stimulates A549 Inflammatory Response

**Figure 2:**
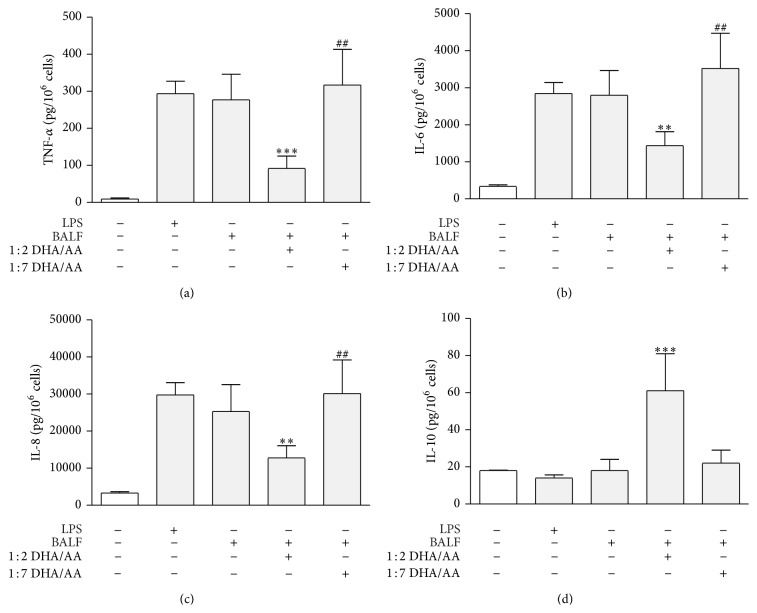
Effects of *ω*-3/*ω*-6 PUFA ratios on BALF induced cytokine release from A549 cells. (a) TNF-*α* proinflammatory cytokine release from A549 cells, stimulated with BALF and treated with 1 : 2 and 1 : 7 DHA/AA PUFA ratios. (b) IL-6 proinflammatory cytokine release from A549 cells stimulated with BALF and treated with 1 : 2 and 1 : 7 DHA/AA (*ω*-3/*ω*-6) PUFA ratios. (c) IL-8 proinflammatory cytokine release from A549 cells stimulated with BALF and treated with 1 : 2 and 1 : 7 DHA/AA PUFA ratios. (d) IL-10 anti-inflammatory cytokine release from A549 cells stimulated with BALF and treated with 1 : 2 and 1 : 7 DHA/AA PUFA ratios. In each panel, data are presented as picograms (pg) of the indicated cytokine per million cells. Data are presented as mean ± standard deviation of 12 independent determinations (*n* = 12). PUFA, polyunsaturated fatty acid; BALF, bronchoalveolar lavage fluid; TNF, tumor necrosis factor; DHA, docosahexaenoic acid; AA, arachidonic acid; IL, interleukin; LPS, lipopolysaccharide. ^***^ *P* < 0.001 1 : 2 DHA/AA versus all. ^**^ *P* < 0.01 1 : 2 DHA/AA versus all. ^##^ *P* < 0.01 1 : 7 DHA/AA versus LPS and BALF.

In a previous study, we demonstrated that human alveolar cells (A549) release proinflammatory cytokines (TNF-*α*, IL-6, and IL-8) in the culture medium when challenged with a proinflammatory stimulus such as LPS, suggesting that the alveolar epithelium has a role in the hyperinflammatory response associated with ARDS [32]. Since LPS is an artificial stimulus, in this study we challenged A549 with BALF of ARDS patients. In this study, BALF stimulation induced a proinflammatory response from A549 cells as demonstrated by release of inflammatory cytokines: TNF-*α* (Figure 2(a)), IL-6 (Figure 2(b)), and IL-8 (Figure 2(c)) while BALF did not elicit anti-inflammatory IL-10 release (Figure 2(d)). These results demonstrated that the pattern of cytokine release of A549 cells exposed to BALF is similar to that elicited by LPS in the previous study.

### 3.3. Opposite Effects of DHA/AA Ratios on the Cytokine Release from A549 Stimulated Cells

The nutrition support of ARDS patients includes lipids, usually soybean or safflower oil-based emulsions. These emulsions contain more than 50% of linoleic acid (*ω*-6), a precursor of AA [42], while are deficient, less than 10%, of *ω*-3 PUFAs (mainly, *α*-linolenic acid). Even though the beneficial effects of *ω*-3 PUFA have been extensively proven by plenty of experimental preclinical data, conflicting results have been obtained from both clinical trials and human intervention studies [15], in ARDS in particular [28]. Therefore, the debate in the scientific community is still open and a definitive accepted recommendation concerning the use of *ω*-3 fatty acids in ARDS is still lacking [42]. The observation that optimal *ω*-3 administration was not only dose-related but was also independently affected by the *ω*-3/*ω*-6 PUFA ratio has led many authors to focus the attention on the *ω*-3/*ω*-6 PUFA ratio in nutrition support to modulate inflammation responses. Different *ω*-3/*ω*-6 PUFA ratios, from 1 : 1 to 1 : 4, have been proposed, but the question of the most favorable *ω*-3/*ω*-6 PUFA ratio in ARDS patients is not definitely established [43].

In a previous study, we demonstrated that, by affecting *ω*-3/*ω*-6 ratio in phospholipids cell membranes with 1 : 1 and 1 : 2 DHA/AA (*ω*-3/*ω*-6) ratio supply, the balance between proinflammatory and anti-inflammatory cytokines was modulated, thus limiting the A549 LPS-induced hyperinflammatory response. Moreover, we found that ratios with a *ω*-6 prevalence (i.e., 1 : 4 and 1 : 7 DHA/AA) potentiated the effect of LPS stimulus [32]. In the present study, we investigated whether 1 : 2 DHA/AA ratio was similarly effective in reducing A549 proinflammatory response induced by an* ex vivo* inflammatory stimulus such as BALF collected from ARDS patients. The 1 : 2 ratio was preferred to 1 : 1 ratio because it could combine efficacy and decreased risk of immunosuppressive effects. The results clearly indicate that 1 : 2 DHA/AA treatment significantly reduced the release of proinflammatory cytokines induced by BALF challenge: TNF-*α* (Figure 2(a)), IL-6 (Figure 2(b)), and IL-8 (Figure 2(c)). Besides, we found that 1 : 2 DHA/AA treatment was also associated with an increased release of the IL-10 (Figure 2(d)), a potent anti-inflammatory cytokine, confirming that 1 : 2 DHA/AA was able to modulate the balance between proinflammatory and anti-inflammatory cytokines. Finally, the 1 : 7 DHA/AA ratio significantly potentiated the BALF inflammatory effects.

Several studies have identified two main key elements in the pathogenesis of ARDS: the occurrence of an imbalance between proinflammatory and anti-inflammatory cytokines [12] and the persistent elevation of proinflammatory mediators [8]. These conditions lead to additional nonpulmonary organ dysfunction which contributes to excess mortality rates in intensive care units [33, 42]. Therefore, strategies for limiting the intensity of lung inflammatory response are of major importance for prognosis and therapy. However, although some pharmacological strategies have proven to be successful in animal studies, human translation of these results has not been so effective on outcome [44].

### 3.4. Biochemical and Molecular Mechanisms Involved in Anti-Inflammatory Effects of *ω*-3 in BALF-Stimulated A549 Cells

**Figure 3:**
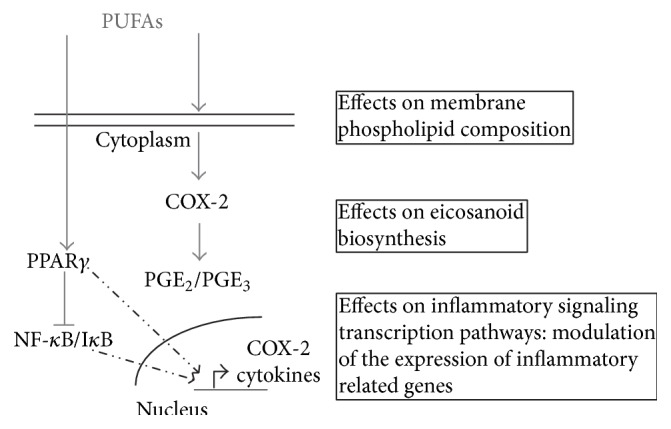
Schematic representation of PUFA mechanism of action in BALF-stimulated A549 cells. PUFAs, polyunsaturated fatty acids; COX, cycloxygenase; PG, prostaglandin; PPAR, peroxisome proliferator-activated receptor; NF-*κ*B, nuclear factor-kappa B; I*κ*B, inhibitor of NF-*κ*B.

Over the last 25 years, the pathophysiology and pharmacology of *ω*-3 PUFA have been continuously under scrutiny. These FA are able to partly inhibit a number of aspects of inflammation including eicosanoid and cytokine production and bioavailability. The PUFA capacity to modulate different signaling pathways involved in inflammation response has been extensively studied in both physiological and pathological conditions [14, 16]. The main mechanisms explaining the PUFA role in inflammation and investigated in this study were graphically represented in Figure 3: effects on phospholipid composition of A549 cell membranes, modulation of eicosanoid and cytokine biosynthesis and release, and effects on inflammatory signaling transcription pathways.

#### 3.4.1. Eicosanoid Synthesis

The modulation of eicosanoid production is driven by modification of the FA composition of the phospholipids within cell membranes. PUFAs are rapidly incorporated into cell membrane phospholipids; moreover, the esterification of *ω*-3 FAs is mainly at the expense of *ω*-6 AA [14]. Membrane phospholipids PUFAs are precursors of eicosanoids, which are the biologically active lipid mediators of inflammation playing wide ranging roles in inflammation and in regulation of immune function [14]. Eicosanoids originated from AA *ω*-6 PUFA (2-series prostaglandins (PGs) and 4-series leukotrienes (LTs)) have proinflammatory properties while eicosanoids formed from EPA and DHA *ω*-3 PUFA (3-series PGs and 5-series LTs) are less active and potentially anti-inflammatory [14]. The balance between proinflammatory and anti-inflammatory eicosanoid synthesis determines the extent of inflammatory reaction [19].

**Figure 4:**
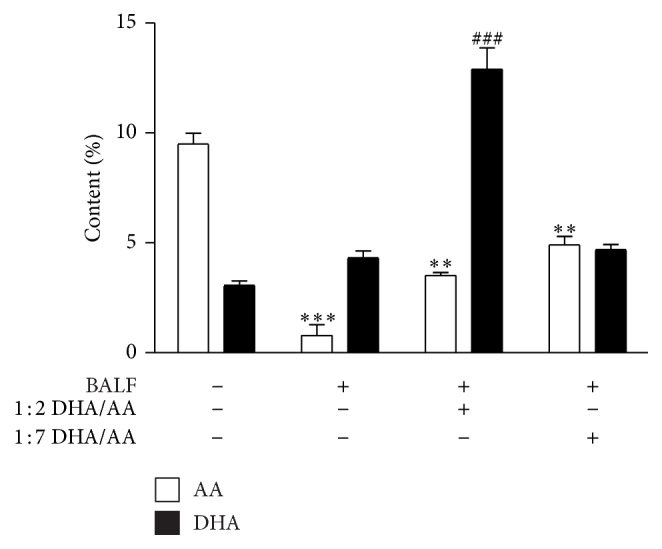
Effects of *ω*-3/*ω*-6 PUFA ratios on the percentage content of AA and DHA in A549 membrane phospholipids. Relative percentage content of AA (white bars) and DHA (black bars) in phospholipids of A549 cell membranes stimulated with BALF and treated with 50 *μ*M 1 : 2 or 1 : 7 DHA/AA PUFA ratios. Data are presented as percentage content of AA and DHA in the membrane phospholipids of unstimulated or stimulated A549 cells as indicated. Data are presented as mean ± standard deviation of 4 independent experiments (*n* = 4). PUFA, polyunsaturated fatty acid; AA, arachidonic acid; DHA, docosahexaenoic acid; BALF, bronchoalveolar lavage fluid. ^***^ *P* < 0.001 BALF versus unstimulated. ^**^ *P* < 0.01 1 : 2 and 1 : 7 DHA/AA versus all. ^###^ *P* < 0.001 versus all.

In our previous study, we demonstrated that at the baseline the *ω*-3/*ω*-6 PUFA ratio in membrane phospholipids of A549 was 1 : 5 and that it can be changed by challenging the cells with 1 : 1 or 1 : 2 DHA/AA ratios [32]. Here, we examined the DHA and AA content in membrane phospholipids of A549 BALF-stimulated and challenged with 1 : 2 or 1 : 7 ratios DHA/AA (Figure 4). At baseline in A549 membrane phospholipids the *ω*-6 PUFA fraction is predominant, with a *ω*-3/*ω*-6 ratio of 1 : 5. Notably, cell treatment with both DHA/AA ratios reduced the AA content in A549 membrane phospholipids, but the 1 : 2 DHA/AA ratio also significantly increased the DHA percentage content. The supply of 1 : 2 DHA/AA ratio reversed the baseline predominance of *ω*-6 over *ω*-3 in the *ω*-3/*ω*-6 PUFA ratio in membrane phospholipids of A549 cells.

Eicosanoid biosynthesis begun when an inflammatory stimulus activates phospholipases, which are the enzymes that cleave the fatty acid precursors from the membrane phospholipids. Released PUFAs are then converted into eicosanoids, mainly PGs and tromboxanes by cycloxygenases (COXs) and LTs by lipoxygenases (LOXs) [14].

There are two COX isoforms: the COX-1 that is constitutively expressed in almost all cells and the COX-2 that is induced in many cell types by a broad range of proinflammatory agents [45]. The COX-2, also known as PG-endoperoxide synthase, is the key enzyme in PG synthesis from AA.

**Figure 5:**
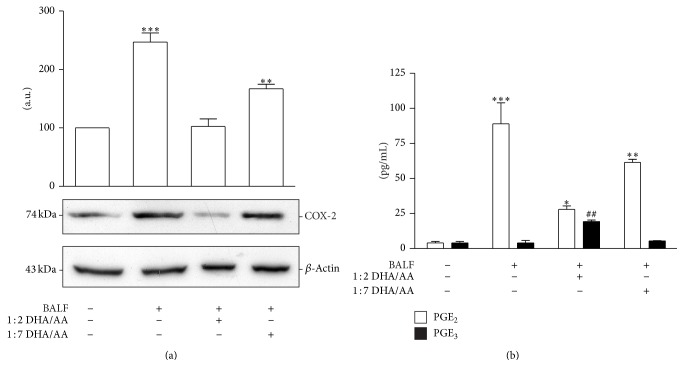
Effects of *ω*-3/*ω*-6 PUFA ratios on PGE_2_ and PGE_3_ synthesis and release. (a) COX-2 relative protein content in A549 cells, stimulated with BALF and treated with 50 *μ*M 1 : 2 and 1 : 7 DHA/AA PUFA ratios. Data are expressed as “a.u.” (arbitrary units) of the densitometric values, normalized on the corresponding *β*-actin. The value of unstimulated cells was arbitrarily set as 100. Data are presented as mean ± standard deviation of 6 independent determinations (*n* = 6). The image is representative of all the WB experiments. (b) PGE_2_ (white bars) and PGE_3_ (black bars) content in culture media of A549 cells, stimulated with BALFs and treated with 50 *μ*M 1 : 2 or 1 : 7 DHA/AA ratio. Data are presented as picograms (pg) of the indicated PG per mL. Data are presented as mean ± standard deviation of 12 independent determinations (*n* = 12). PUFA, polyunsaturated fatty acid; PG, prostaglandin; COX, cycloxygenase; BALF, bronchoalveolar lavage fluid; DHA, docosahexaenoic acid; AA, arachidonic acid; WB, western blot. ^***^ *P* < 0.001 BALF versus unstimulated cells and 1 : 2 DHA/AA. ^**^ *P* < 0.01 1 : 7 DHA/AA versus all. ^*^ *P* < 0.05 1 : 2 DHA/AA versus unstimulated cells. ^##^ *P* < 0.01 1 : 2 DHA/AA versus all.

To verify whether in our experimental model there was a modulation of PG biosynthesis, we analyzed the content of COX-2. The results indicated that in response to BALF stimulus A549 cells increased by 2.5-fold the expression of COX-2 (Figure 5(a)) and produce a significant amount of PGE_2_ (Figure 5(b)). Noteworthy, even if COX-2 is the inducible form of COX, A549 cells express the enzyme constitutively [46]. According to Yang et al. [46], we found that this level of COX-2 expression was not associated with a PG synthesis (Figure 5(b)) in unstimulated A549.

The 1 : 2 DHA/AA treatment significantly restored the expression of the enzyme at the level of unstimulated cells (Figure 5(a)). This effect was significantly associated with a reduction of PGE_2_ release and an induction of PGE_3_ synthesis (Figure 5(b)), suggesting that the enzyme was in an active form. The 1 : 7 DHA/AA ratio induced a less significant inhibitory effect on the COX-2 content and the PGE_2_ synthesis. The differences in COX-2 content could be correlated with the PGE_2_-dependent amplification of the enzyme [45]. These results indicate that both *ω*-3 and *ω*-6 PUFAs reduce the expression of COX-2 induced by inflammatory stimuli such as BALF but with a different extent. These effects on PGE_2_ content well correlated with the availability of AA substrate into membrane phospholipids (Figure 4). Moreover, treatment with 1 : 2 DHA/AA also induced a significant increase in PGE_3_ content, a less potent inflammatory PG. This finding well correlated with the increased DHA content in A549 membrane phospholipids (Figure 4). Notably, PGE_3_ is a less potent inducer of COX-2 gene expression in fibroblasts and of IL-6 production by macrophages compared with PGE_2_ [13]. In accordance with Yang et al.'s data [46], our results confirm that exposure of alveolar cells to *ω*-3 PUFA determines a decrease in the COX-2-mediated formation of PGE_2_ and an increase in the level of PGE_3_.

#### 3.4.2. Inflammatory Signaling Transcription Pathways: NF-*κ*B and PPAR*γ*

**Figure 6:**
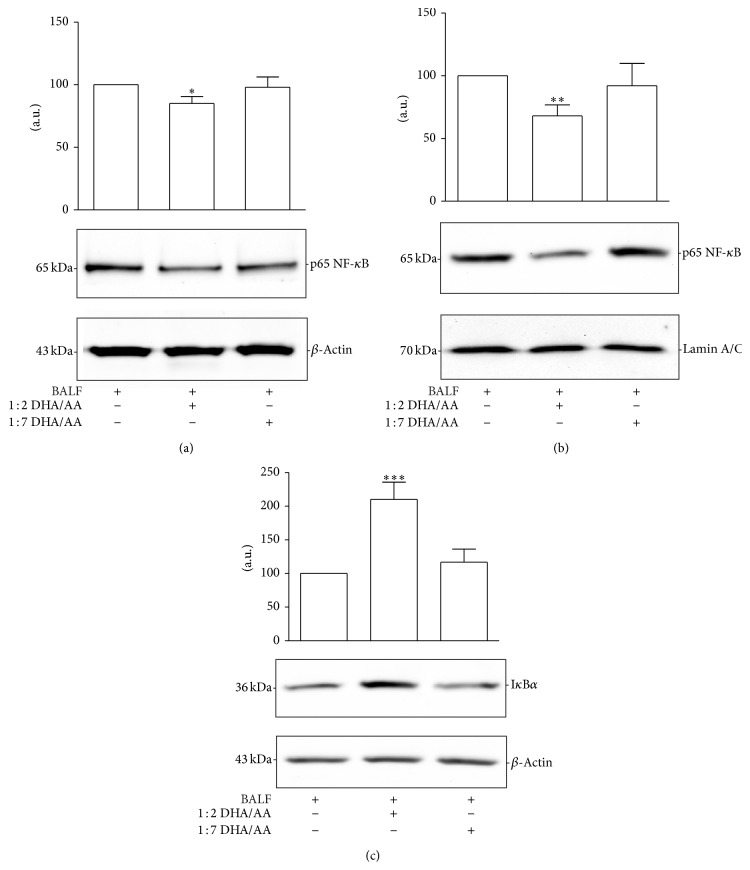
Effects of *ω*-3/*ω*-6 PUFA ratios on NF-*κ*B. (a) p65 NF-*κ*B relative protein content in the cytoplasmic fraction of A549 cells, stimulated with BALF and treated with 50 *μ*M 1 : 2 or 1 : 7 DHA/AA ratios. Data are expressed as “a.u.” (arbitrary units) of the densitometric values, normalized on the corresponding *β*-actin. The value of BALF was arbitrarily set as 100. Data are presented as mean ± standard deviation of 6 independent determinations (*n* = 6). The image is representative of all the WB experiments. (b) NF-*κ*B relative protein content in the nuclear fraction of A549 cells, stimulated with BALF and treated with 50 *μ*M 1 : 2 or 1 : 7 DHA/AA ratios. Data are expressed as “a.u.” (arbitrary units) of the densitometric values, normalized on the corresponding lamin A/C. The value of BALF was arbitrarily set as 100. Data are presented as mean ± standard deviation of 6 independent determinations (*n* = 6). (c) I*κ*B*α* relative protein content in the cytoplasmic fraction of A549 cells, stimulated with BALF and treated with 50 *μ*M 1 : 2 or 1 : 7 DHA/AA ratios. Data are expressed as “a.u.” (arbitrary units) of the densitometric values, normalized on the corresponding *β*-actin. The value of BALF was arbitrarily set as 100. Data are presented as mean ± standard deviation of 6 independent determinations (*n* = 6). The image is representative of all the WB experiments. PUFA, polyunsaturated fatty acid; NF-*κ*B, nuclear factor-kappa B; BALF, bronchoalveolar lavage fluid; DHA, docosahexaenoic acid; AA, arachidonic acid; WB, western blot; I*κ*B, inhibitor of NF-*κ*B. ^*^ *P* < 0.05 1 : 2 DHA/AA versus all. ^**^ *P* < 0.01 1 : 2 DHA/AA versus all. ^***^ *P* < 0.001 1 : 2 DHA/AA versus all.

The nuclear factor (NF-*κ*B) is a key transcription factor involved in upregulation of inflammatory cytokines, adhesion molecules, and COX-2 genes [47]. Activation of NF-*κ*B transcription factor has been implicated in a number of inflammation-related pathologies [14]. The p65 and p50 NF-*κ*B heterodimers are maintained inactive in the cytosol by the binding with an inhibitory protein, namely, inhibitor of NF-*κ*B (I*κ*B) [48]. Proinflammatory stimuli induce phosphorylation, ubiquitination, and proteasome mediated degradation of I*κ*B, allowing NF-*κ*B translocation into the nucleus and NF-*κ*B target gene transcription [48]. The inhibitory role of *ω*-3 PUFAs on NF-*κ*B pathway has been demonstrated in several experimental models and pathological conditions [19]. Different authors demonstrated that *ω*-3 decreased TNF-*α* expression through the prevention of NF-*κ*B activation by inhibiting I*κ*B phosphorylation and consequently preventing NF-*κ*B translocation into the nucleus [14]. The correlation between NF-*κ*B activation and cytokine content in BALF of ARDS patients was previously demonstrated by Nys et al. [49]. In order to understand the role of 1 : 2 DHA/AA treatment on the NF-*κ*B pathway, we analyzed the expression of p65 NF-*κ*B both in the cytoplasmic and the nuclear fraction of A549 BALF-stimulated cells (Figures 6(a) and 6(b)). The results demonstrated that 1 : 2 DHA/AA treatment decreased NF-*κ*B content, in the nuclear fraction in particular, indicating an inhibition of its activation. The results were confirmed by the finding that 1 : 2 DHA/AA treatment increased the content of I*κ*B*α*, one of the isoforms of I*κ*B (Figure 6(c)). These results suggested that the anti-inflammatory effects of 1 : 2 DHA/AA treatment were associated with the inhibition of the NF-*κ*B inflammatory pathway. Since, genes of IL-1*β*, IL-6, and TNF-*α*, as well as COX-2, are regulated by NF-*κ*B [50], we can speculate that the reduction of proinflammatory cytokine release and the inhibition of COX-2 expression were mediated by the inhibition of NF-*κ*B transcriptional activity.

**Figure 7:**
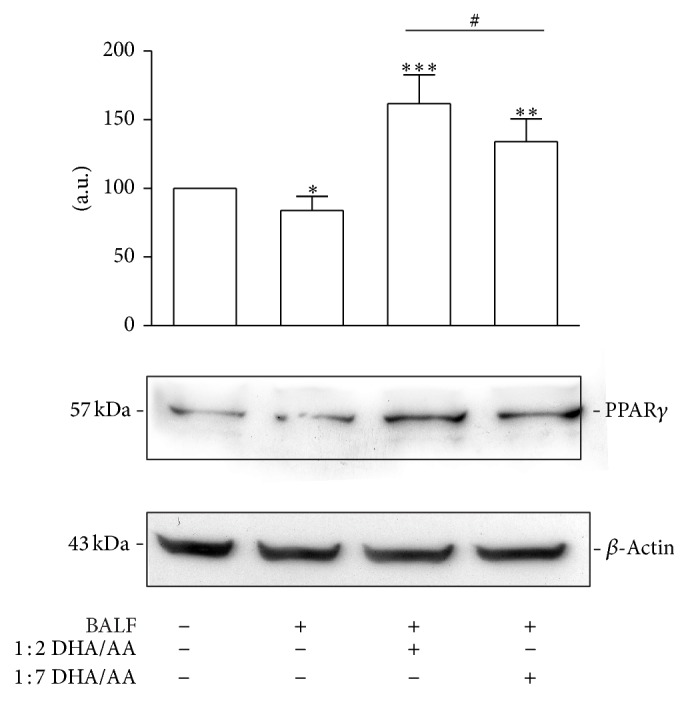
Effects of *ω*-3/*ω*-6 PUFA ratios on PPAR*γ* expression. PPAR*γ* relative protein content in A549 cells stimulated with BALF and treated with 50 *μ*M 1 : 2 or 1 : 7 DHA/AA ratios. Data are expressed as “a.u.” (arbitrary units) of the densitometric values, normalized on the corresponding *β*-actin. The value of unstimulated cells was arbitrarily set as 100. Data are presented as mean ± standard deviation of 6 independent determinations (*n* = 6). The image is representative of all the WB experiments. PUFA, polyunsaturated fatty acid; PPAR, peroxisome proliferator-activated receptor; BALF, bronchoalveolar lavage fluid; DHA, docosahexaenoic acid; AA, arachidonic acid; WB, western blot. ^*^ *P* < 0.05 BALF versus unstimulated cells. ^**^ *P* < 0.01 1 : 7 DHA/AA versus BALF and unstimulated cells. ^***^ *P* < 0.001 1 : 2 DHA/AA versus BALF and unstimulated cells. ^#^ *P* < 0.05 1 : 2 DHA/AA versus 1 : 7 DHA/AA.

Peroxisome proliferator-activated receptors (PPARs) are ligand-activated nuclear transcription factors encoded by different genes. PPARs include 3 subtypes (*α*, *β*, and *γ*), which are characterized by unique functions such as ligand specificities and tissue distribution [51]. PPAR ligands encompass endogenous metabolites such as prostanoids and PUFAs, as well as synthetic drugs such as fibrates and thiazolidinediones. In macrophages, activation of PPAR*γ* negatively influences the production of inflammatory cytokines like TNF-*α*, IL-6, and IL-1*β* [52]. It has been demonstrated that most of the effects of PPARs on cytokine expression result from crosstalk with other transcriptional factors and in particular with NF-*κ*B [53]. To verify if also in our experimental model the anti-inflammatory effects of *ω*-3 were correlated with PPAR activation, we determined the PPAR*γ* content (Figure 7). The results indicated that the proinflammatory stimulus was associated with the inhibition of PPAR*γ* expression. Moreover, as we previously demonstrated [35], in the present study we found both PUFA ratios were associated with an increased PPAR*γ* content but to a greater extent with the 1 : 2 DHA/AA treatment.

Summarizing, we speculate that in our experimental model the anti-inflammatory effects of 1 : 2 DHA/AA treatment could be mediated by reduction of COX-2 expression, decrease of NF-*κ*B translocation into the nucleus, and PPAR*γ* activation. Thus, the results presented herein give further insight into the mechanisms involved in the anti-inflammatory effect of *ω*-3 PUFAs.

#### 3.4.3. Resolvins and Protectins

Recent studies have identified n-3 PUFAs as precursors of a distinct set of lipid mediators that probably act through distinct receptors to exert their anti-inflammatory effects. These new n-3 PUFA-derived, anti-inflammatory mediators have been named resolvins and protectins. For an understanding of resolvin and protectin formation see Weylandt et al. [54] for a complete review. Briefly, these lipid mediators might offer an important new concept to explain the protective effect of n-3 PUFAs in a wide variety of disease models. In particular, DHA constitutes the origin for the D-series resolvins—mainly, resolvin D1 (RvD1)—as well as (neuro-) protectin D1.

In this study, we did not investigate resolvins or protectins in our experimental model; however, previous experimental studies showed that RvD1 had potent anti-inflammatory effects in several disease models including lung injury. RvD1 could modulate the balance between proinflamatory and anti-inflammatory cytokines, alter the response of the host to pulmonary bacterial infection, and affect the early outcome of infection [55]. Wang et al. showed that RvD1 improved survival rate and attenuated ALI in mice induced by LPS; specifically, RvD1 inhibited increases in TNF-*α* and IL-6 production in the BALF, reduced expression of COX-2, and inhibited activation of NF-*κ*B [56].

## 4. Conclusions

ARDS is an inflammatory disease whose clinical severity mostly depends on the grade of inflammation. Cytokines and eicosanoids are key elements in the pathogenesis and outcome of ARDS.

To the best of our knowledge, this is the first study reporting the role of *ω*-3 to *ω*-6 PUFA ratio in the modulation of release of four cytokines (TNF-*α*, IL-6, IL-8, and IL-10) and two prostaglandins (PGE_2_ and PGE_3_) in human alveolar cells exposed to BALF of ARDS patients. Differently from our earlier study, the present study has some original features. First, we challenged alveolar cells with an* ex vivo* inflammatory stimulus and not with LPS. Second, the number of experiments was greater (12 versus 4). Third, we also investigated the PG release as well as the content of COX-2. Finally, we examined the more important inflammatory signal transduction pathways (NF-*κ*B and PPAR*γ*) at work in this experimental inflammatory cell model.

The results of this study demonstrated that shifting the PUFA supply from *ω*-6 to *ω*-3 decreased the release of proinflammatory cytokines and PGE_2_ in human alveolar cells challenged with BALF of ARDS patients. Moreover, these data confirmed our previous finding that a predominance of AA in PUFA supply determined a more aggressive proinflammatory response. Finally, these data provide a contribution to support the biochemical basis for current recommendations [57–60] to shift the lipid supply from *ω*-6 to *ω*-3 PUFA in the nutrition support of ARDS patients.

In conclusion, there are good experimental evidence and convincing rationale according to the *ω*-3 PUFA use in ARDS patients [61]; however, questions still remain to be answered regarding the* in vivo* effects of these PUFAs.
